# The radiomics nomogram predicts the prognosis of pancreatic cancer patients with hepatic metastasis after chemoimmunotherapy

**DOI:** 10.1007/s00262-024-03644-2

**Published:** 2024-03-30

**Authors:** Wenxin Lu, Guangyu Wu, Xianyuan Miao, Jingyu Ma, Yanling Wang, Haiyan Xu, Daiyuan Shentu, Shengbai Xue, Qing Xia, Yu Wang, Liwei Wang

**Affiliations:** 1 Department of Oncology, Ren Ji Hospital, Shanghai Jiao Tong University School of Medicine, Shanghai, 200127 China; 2 Department of Radiology, Ren Ji Hospital, Shanghai Jiao Tong University School of Medicine, Shanghai, 200127 China; 3 Department of Oncology, Ning Bo Hangzhou Bay Hospital, Ningbo, 315336 China; 4 State Key Laboratory of Systems Medicine for Cancer of Shanghai Cancer Institute, Ren Ji Hospital, Shanghai Jiao Tong University School of Medicine, Shanghai, 200127 China

**Keywords:** Pancreatic cancer, Radiomics, Nomogram, Survival analysis, Immunotherapy

## Abstract

**Objective:**

To construct a prognostic model based on MR features and clinical data to evaluate the progression free survival (PFS), overall survival (OS) and objective response rate (ORR) of pancreatic cancer patients with hepatic metastases who received chemoimmunotherapy.

**Methods:**

105 pancreatic cancer patients with hepatic metastases who received chemoimmunotherapy were assigned to the training set (*n* = 52), validation set (*n* = 22), and testing set (*n* = 31). Multi-lesion volume of interest were delineated, multi-sequence radiomics features were extracted, and the radiomics models for predicting PFS, OS and ORR were constructed, respectively. Clinical variables were extracted, and the clinical models for predicting PFS, OS and ORR were constructed, respectively. The nomogram was jointly constructed by radiomics model and clinical model.

**Result:**

The ORR exhibits no significant correlation with either PFS or OS. The area under the curve (AUC) of nomogram for predicting 6-month PFS reached 0.847 (0.737–0.957), 0.786 (0.566–1.000) and 0.864 (0.735–0.994) in the training set, validation set and testing set, respectively. The AUC of nomogram for predicting 1-year OS reached 0.770 (0.635–0.906), 0.743 (0.479–1.000) and 0.818 (0.630–1.000), respectively. The AUC of nomogram for predicting ORR reached 0.914 (0.828–1.00), 0.938 (0.840–1.00) and 0.846 (0.689–1.00), respectively.

**Conclusion:**

The prognostic models based on MR imaging features and clinical data are effective in predicting the PFS, OS and ORR of chemoimmunotherapy in pancreatic cancer patients with hepatic metastasis, and can be used to evaluate the prognosis of patients.

**Supplementary Information:**

The online version contains supplementary material available at 10.1007/s00262-024-03644-2.

## Introduction

In the United States, the incidence rate of pancreatic cancer (PC) ranks 10th among men and 7th among women. The five-year survival rate is only 12% [1]. Pancreatic ductal adenocarcinoma (PDAC) is the most common pathological type of pancreatic cancer. Most PDAC patients are accompanied by distant metastasis when diagnosis [2]. Compared to other sites of metastasis, patients with hepatic metastasis have the worst prognosis, most deaths of PDAC patients are caused by hepatic metastasis [3, 4]. PDAC patients with hepatic metastases have a shorter survival time of only 3–6 months [5].

Chemotherapy is still the main treatment for distant metastatic PDAC. Unlike other cancers, the effect of immunotherapy in PDAC is discouraging. In addition to Paberizumab, immunotherapy is usually ineffective in monotherapy of pancreatic cancer. Even among MSI-H pancreatic cancer, in the KEYNOTE 158 study, pembrolizumab monotherapy only got ORR of 18.2%, mPFS of 2.1 months and mOS of 4.0 months [6]. The preferred regimen for first-line therapy recommended by NCCN guideline for pancreatic cancer with distant metastasis contain FOLFIRINOX regimen, gemcitabine + albumin-bound paclitaxel (AG) regimen, and the only immunotherapy– pembrolizumab (if MSI-H, dMMR, or TMB-H [≥ 10 mut/Mb])[7]. Fortunately, the addition of immunotherapy to chemotherapy shows improved efficacy. In a single-center phase Ib/II study, 11 chemotherapy-naive metastatic PC patients receiving pembrolizumab combined with AG showed mPFS of 9.1 months and mOS of 15.0 months, and the disease control rate (DCR) was 100% [8]. In a phase II clinical trail on KN046 (PD-L1/CTLA-4 dual antibody) combined with AG regimen for unresectable locally advanced or metastatic pancreatic ductal cancer (KN046-IST-04), the ORR was 50.0%, DCR was 95.5%, and PFS-6 M rate was 62.3% [9].

Despite various treatments, the prognosis of patients with pancreatic cancer is still poor. Chemotherapy is characterized by its rapid onset and swift control of the lesion, whereas immunotherapy exhibits a relatively slower response, a response rate but offers a more durable efficacy for specific patients [10]. Simultaneously, we have observed that the rapid onset of chemotherapy does not directly correlate with the extension of the PFS and OS. Given the distinct characteristics of chemotherapy and immunotherapy, the prediction of prognosis in pancreatic cancer patients receiving chemoimmunotherapy becomes increasingly intricate and challenging.

Radiomics refers to the extraction and analysis of quantitative imaging features from computed tomography (CT), magnetic resonance imaging (MRI) or positron emission tomography/X-ray computed tomography (PET/CT) using advanced computational algorithms. It has gained significant attention in the field of oncology, including pancreatic cancer [11, 12]. The prediction of tumor phenotype, treatment response and patient prognosis is becoming feasible by using such comprehensive and integrated radiomics models [13]. Radiomics has shown outstanding predicting ability in immunotherapy for several malignant tumors, and achieved good results in predicting treatment-related changes in transgenic mouse model of PDAC [14, 15]. However, to our best knowledge, radiomics model has not been applied in immunotherapy for PDAC in human. Hence, our endeavor is to employ radiomics in order to predict the prognosis of pancreatic cancer patients with hepatic metastasis who undergo a combination of chemotherapy and immunotherapy.

## Materials and methods

### Patients

Our retrospective study was approved by the Institutional Review Committee of Renji Hospital (KY[2019]035). 74 patients diagnosed with PDAC and hepatic metastasis who visited Renji Hospital (Shanghai, P.R. China) from June 2019 to November 2021 were selected and randomly assigned to the training set (*n* = 52) and the validation set (*n* = 22) according to a 7:3 ratio. 31 patients enrolled in the "Prospective one arm exploratory clinical trail on the efficacy and safety of PD-1 antibody SHR-1210 combined with AG in the first-line treatment of metastatic pancreatic cancer" (RenJi Hospital, ClinicalTrials.gov Identifier: NCT04181645) from November 2019 to March 2021 were selected and included in the testing set. These patients all received PD-1 antibody combined with AG in each cycle of 21 days and were followed up to death. The median follow-up time is 14.7 months (range 2.0–41.8 months).

Inclusion criteria: 1. Pathologically confirmed as PDAC with hepatic metastasis; 2. Surgical assessment of unresectable pancreatic cancer; 3. Upper abdomen MR images were taken in Renji Hospital before treatment, and the lesions were clear and measurable; 4. According to the RECIST 1.1 standard [16], pancreatic and hepatic lesions can be used as target lesions and can be clearly measured; 5. Regularly review MR after every 2 cycles of medication and evaluate the efficacy; 6. Clinical information has been collected, including demography characteristics, medical history, laboratory examination, treatment regimen and time, pathological examination and follow-up data.

Exclusion criteria: 1. Received other anti-tumor treatment before; 2. Patients with hepatic metastasis who can be surgically resectable or borderline resectable; 3. The date of laboratory or MRI examination before treatment exceeds 1 month before treatment; 4. Merging primary malignant tumors from other sites; 5. The image is unclear, or there are no measurable target lesions in the pancreas or liver; 6. Irregular follow-up or incomplete clinical data.

The clinical data and imaging semantic features of patients are obtained from medical records, containing age, gender, primary site of pancreatic cancer, hypertension, diabetes, carbohydrate antigen 19-9 (CA19-9), antigen identified by monoclonal antibody Ki-67 (Ki-67), pancreatic duct obstruction, bile duct obstruction, number of hepatic metastases, maximum diameter of hepatic lesion, MR-reported T stage, MR-reported N stage, neutrophil-to-lymphocyte ratio (NLR) and platelet-to-lymphocyte ratio (PLR). TNM stage for all patients is judged based on American joint committee on cancer (AJCC) TNM staging criteria for pancreatic cancer 8th edition [17]. The evaluation of imaging efficacy is based on the RECIST 1.1 standard. Patients with based on imaging finding partial response (PR) or complete response (CR), are classified as "objective response" patients. Patients with stable disease (SD) or progressive disease (PD) are classified as "non-objective response" patients. The patient's imaging data was independently evaluated by 2 radiologists who worked for 9 and 11 years, respectively, without knowing the patient's clinical information.

### Imaging protocol

MRI was performed according to a standard protocol using a 3.0 T permanent field (Ingenia, Philips, Netherlands) [18]. The detailed MRI protocol was listed in table S8. Image preprocessing includes N4 bias field correction and histogram standardization of MRI scan intensity values [19].

Sequences including T1WI, T2WI and DWI were first preprocessing with IntelliSpace Portal (Philips Healthcare, Best, Netherlands). 2 tumor regions were selected: the lesion of primary tumor in pancreas and one of the largest measurable metastatic lesions in liver. Three-dimensional VOI of the pancreatic lesion and the hepatic lesion were manually delineated slice-by-slice using the ITK-SNAP software by a radiologist (Wu GY with 13 years’ experience in abdominal imaging). The tumor contours were primarily delineated on concurrent T2-weighted images where DWI/ADC images were used for cross-check and finetune. To select robust features against intra-rater and inter-rater delineation variations, the VOI delineation process was repeated by the same radiologist and by another radiologist (Liu GQ with 12 years’ experience in abdominal imaging), yielding an intra-rater test data set and an inter-rater test data set, respectively.

Features were extracted strictly following the Biomerker Standardization Initiative (IBSI) specification [20]. The features of pancreatic and hepatic regions of interest (ROIs) were extracted with IntelliSpace Portal, containing original shape-based features, first-order statistical features, gray-level cooccurrence matrix (Glcm) features, ray-level run length matrix (Glrlm) features and gray-level size zone matrix (Glszm) features. For each ROI, 550 features of T2WI and DWI sequences were extracted, respectively. Next, we merged features of these 2 sequences into a new dataset named “T2&DWI” features, which contained 1100 joint features in total [21, 22]. Furthermore, we calculated the average of features extracted from pancreatic and hepatic ROIs according to the volume-weighted of the lesions, and obtain a new dataset which amounted to the features of a new “primary&met” ROI [23]. At this point, we have obtained six datasets of features, namely: “T2-primary”, “DWI-primary”, “T2&DWI-primary”, “T2-primary&met”, “DWI-primary&met” and “T2&DWI-primary&met”.

### Statistical analysis

The Z-score standard method was used to normalize all features, so that each feature was on the same order of magnitude.

To construct an radiomics model for predicting PFS and OS, in the training set, a three-stage feature selection method was implemented. Initially, the intra- and inter-observer repeatability of each feature was evaluated using the intraclass correlation coefficient (ICC) on the test–retest and multi-delineation data sets, respectively. Features with low repeatability, indicated by an ICC < 0.75, were excluded. Secondly, the Max-Relevance and Min-Redundancy (mRMR) method is chosen to eliminate redundant and uncorrelated features, thereby retaining 30 features for subsequent selection. Finally, the Cox proportional hazard model with the Least absolute shrinkage and selection operator (Lasso-Cox) model was used to further reduce the feature [24]. To determine the optimal model parameter, tenfold cross-validation (CV) was conducted by calling the "cv.glmnet" function in "glmnet" package. Finally, the features exhibiting non-zero coefficients were chosen, and the radscore was calculated from these selected features to predict PFS and OS. Due to the seed parameter of tenfold CV grouping process being random in the default glmnet package, the output of each function call is different, which may potentially affect the stability and robustness of the feature selection process. To evaluate the stability and robustness, we ran the cv.glmnet function 10 times, outputting 10 regularization parameters (i.e., λ). 10 regressions were conducted using the λ from each CV, and the occurrence frequency of the features selected in 10 regressions was obtained. To evaluate the predictive performance of the radscore in the validation set and the testing set, ROC curve analysis was employed, with the AUC serving as the key metric. Heatmaps are selected to display Standardized feature parameters and radscore values of patients in different PFS or OS groups.

To construct an radiomics model for differentiating patients of objective response, an Linear Discriminant Analysis (LDA) classifier was employed [25, 26]. Baseline images before treatment and reviewed images after 2 cycles of treatment were selected, then normalized the radiomic feature intensities. After excluding the features with ICC < 0.75, the percent change of feature statistics between baseline and reviewed images was calculated to yield the LDA dataset [27]. ROC analysis with AUC was also selected to evaluate the predictive performance of LDA classifier.

**Fig. 1:**
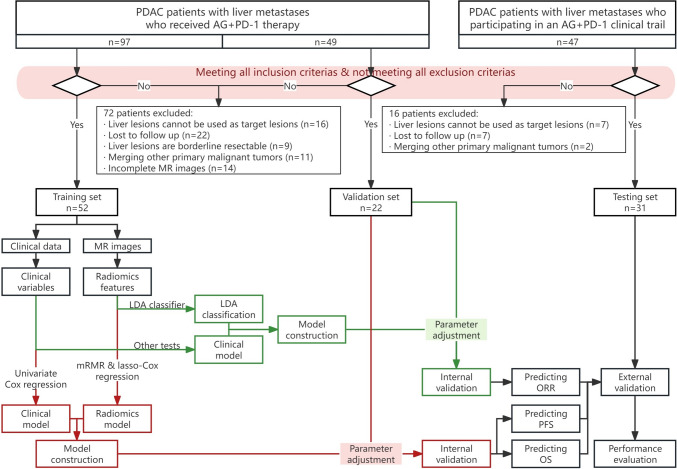
The flowchart of this study

We performed the statistical analysis with SPSS statistical software (version 24.0.0, IBM, USA) and R software (version 4.0.5). The primary endpoints were 6-month PFS, 1-year OS, and ORR. Clinical data related to PFS and OS were analyzed using univariate Cox regression analysis, and the hazard ratios (HR) were calculated. The clinical data related to ORR were tested using the other tests (Table 2). *p* < 0.05 means the significant difference, and variables with *p* < 0.05 were selected and merged with corresponding radscore or LDA classification to form composite predictive models. Nomograms were established based on the composite models. ROC with AUC was employed to evaluate the predictive performance of the models. In order to assess the clinical utility of the nomograms, we conducted a DCA of multivariate Cox regression to compare the net benefits derived from the implementation of nomograms, radscore (or LDA classification), and clinical models [28]. The calibration curve is used to evaluate the calibration of the models [29]. Kaplan-Meire survival analysis was used to analyze the differences in PFS and OS between groups with high and low nomo-scores (Fig. 1).

## Result

### Characteristics of patients

**Fig. 2:**
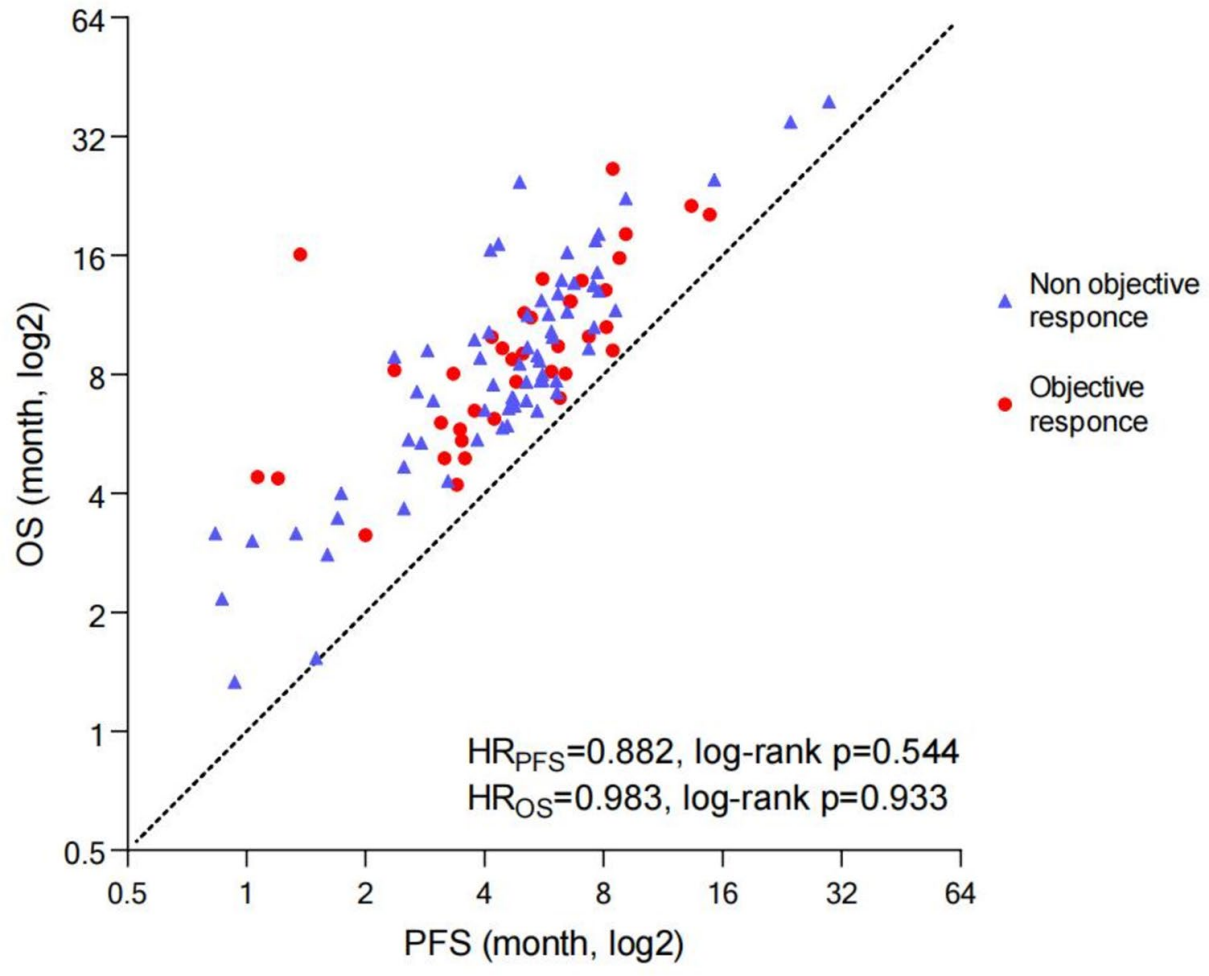
Distribution of PFS, OS and ORR

The median (Q_L_,Q_U_) of PFS in training set, validation set and testing set are 5.12 (3.36, 6.56) months, 5.11 (3.57, 7.30) months and 4.90 (3.58, 6.00) months, respectively, and that of OS are 9.30 (5.90, 11.48) months, 8.73 (6.58, 12.81) months and 8.20 (6.53, 12.92) months, respectively, the difference is not statistically significant by the Log Rank test (*p*_PFS_ = 0.76, *p*_OS_ = 0.96) (Figure S1). The ORR are 44.23%, 27.27% and 29.03%, respectively, the difference is not statistically significant by the Pearson’s chi-square test (*p* = 0.234). Objective response is not correlated with PFS or OS whether in training set, validation set or testing set (all *p* > 0.05) (Fig. 2 & Figure S1).

There were no significant differences in any clinical features between training set, validation set or testing set. The inter-group data has good equivalence (Table 1).

**Table 1:** Baseline characteristics of clinical factors and imaging semantic features of patients in the training set, validation set and the testing set

Variable	Training set (*n* = 52)	Validation set (*n* = 22)	Testing set (*n* = 31)	*p*
Age, mean ± SD	59.62 ± 9.14	61.64 ± 9.12	62.81 ± 7.05	0.206^b^
Gender, *n* (%)				0.975^a^
Male	36 (69.23)	15 (68.18)	22 (70.97)	
Female	16 (30.77)	7 (31.82)	9 (29.03)	
CA-199, median (Q_L_,Q_U_)	1799.5 (733.0,6195.3)	2476.0 (702.8,8207.8)	1197.0 (329.0,3882.0)	0.392^b^
Ki-67, median (Q_L_,Q_U_)	0.3 (0.2,0.5)	0.3 (0.1,0.58)	0.3 (0.1,0.5)	0.997^b^
Primary site of pancreatic cancer, *n* (%)				0.857^a^
Head & neck	18 (34.62)	9 (40.91)	12 (38.71)	
Body & tail	34 (65.38)	13 (59.09)	19 (61.29)	
Hypertension, *n* (%)				0.589^a^
Yes	10 (19.23)	5 (22.73)	9 (29.03)	
No	42 (80.77)	17 (77.27)	22 (70.97)	
Diabetes, *n* (%)				0.711^a^
Yes	11 (21.15)	5 (22.73)	9 (29.03)	
No	41 (78.85)	17 (77.27)	22 (70.97)	
Pancreatic duct obstruction, *n* (%)				0.250^a^
Yes	17 (32.69)	11 (50.00)	9 (29.03)	
No	35 (67.31)	11 (50.00)	22 (70.97)	
Bile duct obstruction, *n* (%)				1.000^a^
Yes	8 (15.38)	3 (13.64)	4 (12.90)	
No	44 (84.62)	19 (86.36)	27 (87.10)	
Number of hepatic metastases, *n* (%)				0.698^b^
1–2	8 (15.38)	2 (9.09)	4 (12.90)	
3–5	4 (7.69)	3 (13.64)	6 (19.35)	
> 5	40 (76.92)	17 (77.27)	21 (67.74)	
Maximum diameter of hepatic lesion, mean ± SD	26.31 ± 11.08	33.23 ± 12.87	29.68 ± 10.68	0.052^a^
MR-reported T stage, *n* (%)				0.843^b^
T1	3 (5.77)	0 (0.00)	1 (3.23)	
T2	7 (13.46)	6 (27.27)	6 (19.35)	
T3	25 (48.08)	8 (36.36)	16 (51.61)	
T4	17 (32.69)	8 (36.36)	8 (25.81)	
MR-reported N stage, *n* (%)				0.910^b^
N0	31 (59.62)	12 (54.55)	19 (61.29)	
N1	2 (3.85)	2 (9.09)	2 (6.45)	
N2	19 (36.54)	8 (36.36)	10 (32.26)	
NLR*, median (Q_L_,Q_U_)	3.53 (2.45,4.25)	2.84 (2.08,4.41)	3.55 (2.73,4.80)	0.426^b^
PLR*, median (Q_L_,Q_U_)	137.1 (109.6,191.3)	143.0 (112.0,188.9)	162.4 (120.8,215.9)	0.518^b^

CA-199, carbohydrate antigen 19–9; Ki-67, antigen identified by monoclonal antibody Ki-67; NLR, Neutrophil-to-lymphocyte ratio; PLR, platelet-to-lymphocyte ratio

^a^Chi-square test or Yates's correction for continuity

^b^Kruskal–Wallis test

### Evaluation of radiomics features

After excluding radiomics features with ICC < 0.75, 341 features in T2WI sequences were selected, containing 10 original shape-based features, 122 first-order features, 124 Glcm features, 51 Glrlm features and 34 Glszm features. 411 features in DWI sequences were selected, containing 9 original shape-based features, 160 first-order features, 147 Glcm features, 62 Glrlm features and 95 Glszm features.

Radscore is an independent variable for predicting PFS and OS. Whether in training set, validation set or testing set, the HR (95%CI) for PFS are 3.21 (1.76–5.85), 1.65 (1.07–2.53) and 2.02 (1.29–3.16), respectively, and the HR (95%CI) for OS are 7.09 (2.34–21.54), 2.71 (1.22–6.03) and 6.70 (1.95–23.07), respectively (all *p* < 0.05). LDA classification is an independent variable for predicting ORR (all *p* < 0.05). In training set, maximum diameter of hepatic lesion is an independent variable for predicting PFS (HR (95% CI) = 1.04 (1.02–1.07), *p* < 0.001), hypertension is an independent variables for predicting objective response (*p* = 0.011), but there is no clinical variable independently predicting OS (all *p* > 0.05) (Table 2).

**Table 2:** Univariate Cox regression analysis of clinical factors of patients in the training set, validation set and the testing set

Variables	Training set (*n* = 52)			Validation set (*n* = 22)			Testing set (*n* = 31)		
	PFS: HR (95% CI), pa	OS: HR (95% CI), pa	ORR: p	PFS: HR (95% CI), pa	OS: HR (95% CI), pa	ORR: p	PFS: HR (95% CI), pa	OS: HR (95% CI), pa	ORR: p
Age	1.00 (0.97–1.04), 0.884	1.02 (0.99–1.06), 0.163	0.680^c^	1.01 (0.96–1.07), 0.597	1.02 (0.97–1.08), 0.482	0.545^c^	1.05 (1.00–1.11), 0.055	0.99 (0.95–1.05), 0.985	0.946^c^
Gender, (male vs female)	0.92 (0.51–1.67), 0.786	0.94 (0.52–1.73), 0.852	0.963^b^	1.34 (0.54–3.38), 0.529	1.24 (0.49–3.10), 0.654	0.674^b^ 0.658^d^	1.59 (0.72–3.50), 0.254	1.63 (0.74–3.62), 0.226	0.922^b^
CA-199	1.00 (1.00–1.00), 0.892	1.00 (1.00–1.00), 0.946	0.719^d^	1.00 (1.00–1.00), 0.261	1.00 (1.00–1.00), 0.141		1.00 (1.00–1.00), 0.076	1.00 (1.00–1.00), 0.372	0.408^d^
Ki-67	2.33 (0.64–8.55), 0.202	1.15 (0.30–4.38), 0.834	0.830^d^	4.23 (0.55–27.50), 0.132	4.79 (0.59–38.79), 0.143	0.159^d^	0.58 (0.13–2.47), 0.458	0.38 (0.08–1.74), 0.210	0.759^d^
Primary site of pancreatic cancer, (Head & neck vs Body & tail)	0.99 (0.55–1.78), 0.969	0.64 (0.36–1.14), 0.132	0.573^b^	1.89 (0.73–4.89), 0.189	2.24 (0.89–5.65), 0.086	0.057^b^	1.03 (0.50–2.15), 0.933	0.53 (0.25–1.13), 0.101	1.000^b^
Hypertension, (yes vs no)	1.15 (0.57–2.33), 0.698	1.25 (0.61–2.56), 0.533	0.011^b^	1.17 (0.45–3.06), 0.749	1.94 (0.72–5.19), 0.189	0.194^b^	1.44 (0.63–3.30), 0.389	1.96 (0.84–4.56), 0.119	0.012^b^
Diabetes, (yes vs no)	1.25 (0.64–2.46), 0.517	1.15 (0.59–2.26), 0.681	1.000^b^	0.59 (0.21–1.69), 0.326	0.67 (0.24–1.86), 0.445	0.572^b^	0.82 (0.36–1.85), 0.630	1.21 (0.53–2.80), 0.549	1.000^b^
Pancreatic duct obstruction, (yes vs no)	0.88 (0.48–1.59), 0.662	0.91 (0.49–1.68), 0.764	0.757^b^	0.70 (0.28–1.79), 0.460	0.51 (0.19–1.33), 0.170	1.000^b^	0.99 (0.44–2.20), 0.979	1.18 (0.53–2.59), 0.687	0.332^b^
Bile duct obstruction, (yes vs no)	0.54 (0.24–1.23), 0.145	0.60 (0.27–1.36), 0.222	0.976^b^	2.21 (0.59–8.26), 0.237	0.96 (0.18–3.32), 0.949	1.000^b^	0.42 (0.14–1.25), 0.119	0.59 (0.20–1.73), 0.338	0.689^b^
Number of hepatic metastases, (1 ~ 2 vs 3 ~ 5 vs > 5)	1.43 (0.95–2.16), 0.089	1.30 (0.88–1.93), 0.189	0.548^d^	1.31 (0.67–2.55), 0.428	1.74 (0.90–3.38), 0.103	0.801^d^	1.41 (0.82–2.42), 0.217	1.44 (0.81–2.54), 0.211	0.342^d^
Maximum diameter of hepatic lesion	1.04 (1.02–1.07), 0.000	1.02 (1.00–1.05), 0.051	0.254^c^	1.05 (1.02–1.09), 0.004	1.03 (1.00–1.06), 0.090	0.122^c^	1.04 (1.00–1.08), 0.023	1.04 (1.01–1.07), 0.019	0.924^c^
MR-reported T stage, (T1–T4)	1.19 (0.85–1.65), 0.308	1.02 (0.74–1.42), 0.899	0.491^d^	1.05 (0.61–1.80), 0.864	0.98 (0.56–1.71), 0.943	0.158^d^	1.67 (1.06–2.66), 0.029	1.45 (0.96–2.18), 0.079	0.123^d^
MR-reported N stage, (N0– N2)	1.04 (0.78–1.39), 0.791	0.93 (0.70–1.25), 0.637	0.460^d^	1.09 (0.69–1.71), 0.727	1.23 (0.78–1.96), 0.377	0.136^d^	0.90 (0.61–1.34), 0.608	0.78 (0.51–1.15), 0.195	0.594^d^
NLR	1.10 (0.94–1.29), 0.240	1.05 (0.88–1.25), 0.600	0.343^d^	0.86 (0.68–1.10), 0.228	0.98 (0.79–1.23), 0.885	1.000^d^	1.01 (0.84–1.21), 0.953	1.07 (0.86–1.33), 0.569	0.486^d^
PLR	1.00 (1.00–1.01), 0.285	1.00 (1.00–1.01), 0.202	0.220^d^	1.00 (0.99–1.00), 0.388	1.00 (0.99–1.01), 0.616	0.161^d^	1.00 (1.00–1.00), 0.381	1.00 (1.00–1.00), 0.733	0.074^d^
Radscore	3.21 (1.76–5.85), 0.000	7.09 (2.34–21.54), 0.001		1.65 (1.07–2.53), 0.023	2.71 (1.22–6.03), 0.014		2.02 (1.29–3.16), 0.002	6.70 (1.95–23.07), 0.003	
LDA classification, (0 vs 1)			0.000^b^			0.008^b^			0.028^b^

CA-199, carbohydrate antigen 19–9; Ki-67, antigen identified by monoclonal antibody Ki-67; NLR, Neutrophil-to-lymphocyte ratio; PLR, platelet-to-lymphocyte ratio

^a^Cox's proportional-hazards model

^b^Chi-square test or Yates's correction for continuity

^c^*T* test

^d^Wilcoxon rank sum test

### Evaluation of radiomics models

Patients in training set and validation set were divided into groups based on whether the PFS/OS was greater than the median. AUC for each of radscores of the six datasets in validation set was calculated (Table 3). The radscore of "T2&DWI-primary&met" dataset has the highest AUC (95% CI) of 0.735 (0.515, 0.955) and 0.743 (0.479, 1.000), respectively, in predicting PFS and OS (Table 3). Radscores show significant differences in predicting PFS and OS both in training set, validation set and testing set (all *p* < 0.05) (Figure S2).

**Table 3:** ROC curve of radscore in predicting PFS and OS in validation set

Dataset	AUC_PFS_ (95%CI)	AUC_OS_ (95%CI)
T2-primary	0.684 (0.454, 0.913)	0.676 (0.426, 0.927)
DWI-primary	0.581 (0.332, 0.831)	0.658 (0.419, 0.897)
T2&DWI-primary	0.718 (0.486, 0.950)	0.638 (0.387, 0.890)
T2-primary & met	0.709 (0.466, 0.953)	0.733 (0.507, 0.960)
DWI-primary & met	0.718 (0.497, 0.939)	0.657 (0.376, 0.938)
T2&DWI-primary & met	0.735 (0.515, 0.955)	0.743 (0.479, 1.000)

Overall, the prediction performance of radscore fitted by features from T2-DWI-combined sequence is better than that of each single sequence, and the performance of features from a multi-lesion ROI are better than that from each single-lesion ROI. However, these differences were not statistically significant (*p* > 0.05). Based on this result, radscores fitted from the “T2&DWI-primary&met” dataset were chosen to construct the radiomics models for predicting PFS and OS.

Radiomics models for predicting PFS and OS both showed stability and robustness. In the PFS model and OS model, the *λ* parameter values output from 10 CV operations were 0.134625 ± 0.0212354 and 0.140412 ± 0.019842, respectively, which indicated a concentrated distribution of λ values. The frequency of the features selected for constructing radiomics models showed to be highly conserved in 10 regressions (Figure S10).

Eventually, the radscore for PFS and OS was, respectively, calculated from 7 features in “T2&DWI-primary&met” dataset, the specific formula and the heatmaps constructed from the radscore and the chosen features of each patient can be found in supplementary materials (Figures S4 & S6).

### Evaluation of nomogram model for PFS

**Fig. 3:**
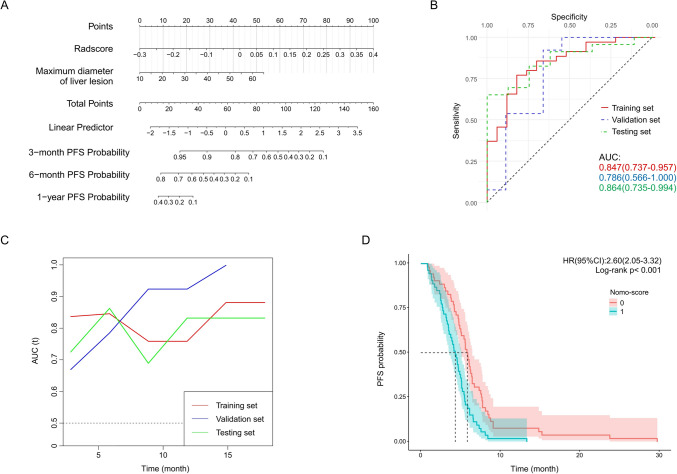
Nomogram for predicting PFS and its evaluation. Nomogram performs well in predicting PFS in training set, validation set and testing set. **A** Nomogram composed of radscore and maximum diameter of hepatic lesion. **B** The ROCs representing the prediction performance of the nomogram in predicting 6-month PFS in training set, validation set and testing set. **C** The time-AUC curves representing the time-correlated prediction performance of the nomogram. **D** Kaplan–Meier analysis between patients with lower nomogram score (group 0) and higher nomogram score (group 1) in training set, validation set and testing set, the HR (95% CI) and log-rank test p value is calculated

According to the univariate Cox regression results in training set, variables chosen to construct nomogram for PFS include radscore and maximum diameter of hepatic lesion (Fig. 3A). The AUC of nomogram for predicting 6-month PFS in training set, validation set and testing set are 0.847 (0.737–0.957), 0.786 (0.566–1.000) and 0.864 (0.735–0.994), respectively (Fig. 3B). In the time-AUC, nomogram shows good predictive performance in predicting PFS (Fig. 3C). PFS between different nomogram-score groups shows significant differences (HR = 2.60, *p* < 0.001) whether in overall patients or in each set (Fig. 3D & Figure S3). The calibration curves of nomogram exhibit good consistency between the prediction and observation of 6-month PFS in all sets (Figure S3). ROC, time-AUC, and DCA analysis suggests that nomogram exhibits the best predictive performance in predicting 6-month PFS compared with radiomics model and clinical model (Figure S3).

### Evaluation of model for OS

**Fig. 4:**
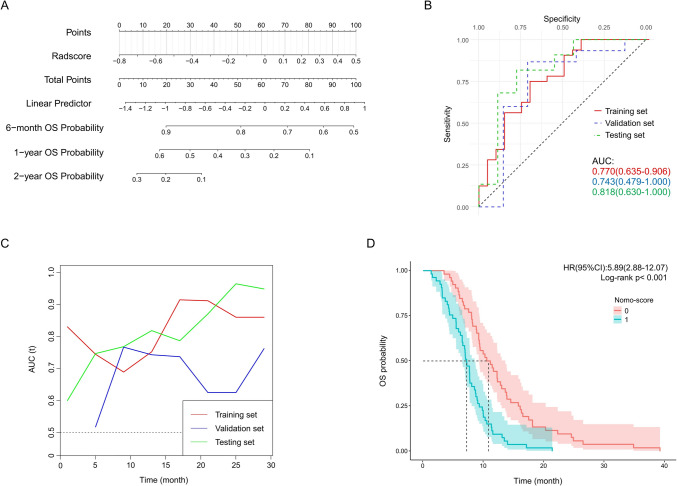
Nomogram for predicting OS and its evaluation. Nomogram performs well in predicting OS in training set, validation set and testing set. **A** Nomogram composed of radscore only. **B** The ROCs representing the prediction performance of the nomogram in predicting 1-year OS in training set, validation set and testing set. **C** The time-AUC curves representing the time-correlated prediction performance of the nomogram. **D** Kaplan–Meier analysis between patients with lower nomogram score (group 0) and higher nomogram score (group 1) in training set, validation set and testing set, the HR (95% CI) and log-rank test p value is calculated

There was no clinical variable to be an independent predictor of OS in training set (Table 2), and the results of a multivariate Cox regression analysis were still negative. Therefore, the nomogram for predicting OS is only constructed from radiomics data (Fig. 4A). The AUC of nomogram for predicting 1-year OS in training set, validation set and testing set are 0.770 (0.635–0.906), 0.743 (0.479–1.000) and 0.818 (0.630–1.000), respectively (Fig. 4B). The model performs well in predicting OS according to the time-AUC (Fig. 4C). OS between different nomogram-score groups shows significant differences (HR = 5.89, *p* < 0.001) whether in overall patients or in each set (Fig. 4D & Figure S5). The calibration curves of nomogram exhibit consistency between the prediction and observation of 1-year OS in all sets (Figure S5). DCA analysis suggests decent value of predicting OS in all sets (Figure S5).

### Evaluation of nomogram model for ORR

**Fig. 5:**
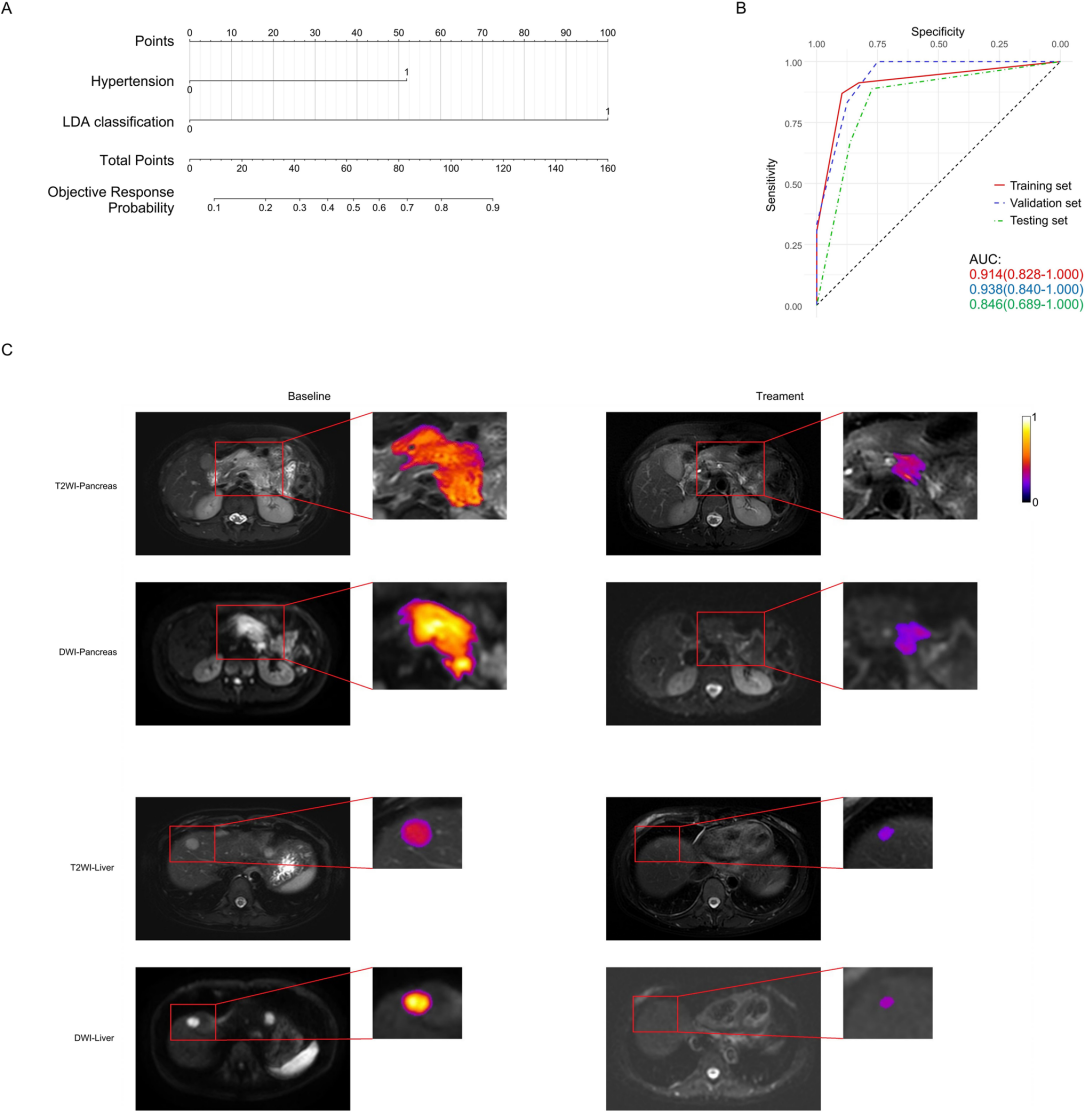
Nomogram for predicting objective response and its evaluation. Nomogram performs well in predicting objective response. **A** Nomogram composed of LDA classification and hypertension. **B** The ROCs representing the prediction performance of the nomogram in predicting objective response in training set, validation set and testing set. **C** MRI responses PR in a patient before and after treatment. The lesion is delineated by an enlarged pseudocolor image

Hypertension is a predictive variable in both the training and testing sets (Table 2). Variables chosen to construct nomogram for objective response include LDA classification and hypertension (Fig. 5A). The AUC of nomogram for predicting objective response in training set, validation set and testing set are 0.91 (0.83–1.00), 0.94 (0.84–1.00) and 0.85 (0.69–1.00), respectively (Fig. 5B). The calibration curves of nomogram exhibit good consistency between the prediction and observation of objective response (Figure S7). In all sets, nomogram shows the highest AUC compared with LDA classification and clinical model (Figure S7). DCA analysis suggests greater value of predicting objective response with nomogram than predicting with LDA classification or clinical model (Figure S7).

## Discussion

The integration of immunotherapy and chemotherapy has instilled renewed optimism in the realm of PDAC treatment, while simultaneously presenting formidable obstacles in achieving accurate treatment and prognosis forecasts [30]. Determining whether chemotherapy or immunotherapy plays a leading role in controlling tumor remains a challenging task in clinical practice. Furthermore, the pseudoprogression effect induced by immunotherapy can potentially confound the imaging evaluation [31]. To address these issues, this study employs predictive models that leverage radiomics parameters and clinical data, exhibiting noteworthy efficacy in forecasting PFS, OS, and ORR among pancreatic cancer patients with hepatic metastasis undergoing chemoimmunotherapy treatment.

Our clinical data reveals an intriguing finding that patients achieving objective response do not necessarily experience extended PFS or OS. In general, patients achieving objective response tends to exhibit a favorable response to chemotherapy, while patients archiving long-term tumor control show a greater likelihood of responding positively to immunotherapy. Radiomics uses quantitative imaging methodological approaches to extract texture features and reflect complex structures that are difficult for imaging doctors to recognize with the naked eyes [32]. The underlying pathological mechanism through which these characteristics impact the efficacy of immunotherapy lies in the compromised vascular structure within tumor tissue, coupled with an abundance of extracellular matrix, impeding the effective penetration and infiltration of immune cells [33]. Furthermore, the tumor microenvironment, characterized by immunosuppression, presents a complex setting where monotherapy proves ineffective [34].

The Tumor microenvironment of PDAC involves the aggregation of fibroblasts. In some patients, the number of stromal cells (including fibroblasts) may exceed that of cancer cells [35]. Therefore, relying solely on changes in tumor size before and after medication is not sufficient to demonstrate the true response of the tumor to the drug. Consequently, by comparing changes in radiomics features before and after medication, we can discern the true response, aiding in accurate identification. The addition of Delta Radiomics has increased the available quantitative information related to intra-tumor heterogeneity (ITH) in space and time, which may reflect the changes of tumor phenotype over time, which is crucial to evaluate the response of immunotherapy [36].

Another common problem encountered in clinical practice involves patients with multiple metastases, where some lesions exhibit a response to anti-tumor therapy while others do not. This discrepancy can be attributed to two key factors: tumor heterogeneity and the pronounced divergence in the immune microenvironment between PDAC metastases and primary diseases, as supported by prior research [37]. Consequently, the RECIST 1.1 standard employs the summation of several representative target lesion diameters as an evaluation criterion, instead of focusing solely on a single target lesion's diameter [16]. Therefore, the radiomics characteristics of an individual lesion alone cannot adequately predict the efficacy and prognosis of patients with metastatic lesions. To address this limitation, in accordance with the RECIST 1.1 standard, we have implemented a volume-weighted calculation method for the features of multiple lesions, a methodology proven to be more effective in previous studies [23]. This study validates that this algorithm, which encompasses multiple lesions in a joint prediction framework, outperforms involving a single lesion alone.

Enhanced MR usually takes longer to schedule than MR plain scan and is more expensive. T2WI and DWI were turn out to be more important in predicting treatment outcomes in this study. T2WI and DWI are considered to be associated with the property and movement of water molecules. T2WI relies upon the transverse relaxation (also known as "spin–spin" relaxation) of the net magnetization vector (NMV), which is related to the rate of rotation and translation of the water molecule or adjacent dipoles. While DWI is a sequence capable of capturing the diffusion and restriction of water molecules within tissues and lesions and always be linked with cell density, molecular distribution, and permeability et al. [38]. The research conducted by Zhang et al. [39]. suggests that an radiomics model constructed by combining multiple sequences outperforms the predictive capacity of a single sequence. Our own investigation corroborates this finding, as the features combined T2 with DWI sequence yield an improved radscore for predictive performance compared to using T2 and DWI features independently. These results demonstrate the good performance of MR plain scan in prognosis prediction, and present novel insights for expanding the repertoire of radiomics prediction model methodologies.

LDA, also known as Fisher discriminant analysis, was originally proposed by Fisher in 1936 as a dimensionality reduction technique for supervised learning to solve binary classification problems. It determines a subspace in which the between-class scatter is as large as possible, while the within-class scatter is kept constant. In this sense, the subspace obtained by LDA optimally discriminates the classes-faces. It initially shone brightly in the field of image recognition. Due to the discriminative nature of LDA, it can accurately perform dimensionality reduction classification [40]. Therefore, more and more scholars are applying LDA classifiers in the medical field, such as disease identification, omics data dimensionality reduction, molecular typing, etc. In this study, it was a supervised learning process with a binary outcome to predict whether objective response had been achieved. In addition to LDA, classical supervised binary classification algorithms include Logistic Regression (LR), Support Vector Machine (SVM), K-nearest Neighbor algorithm (K-NN), Decision Tree, Naive Bayes, etc. LDA has shown performance comparable to other classifiers in various radiomics studies [41, 42]. Noto et al. used cardiac MRI to distinguish myocardial infarction and myocarditis, and found that LDA using 3D features produced significantly higher discrimination results than K-NN, SVM, and TreeBagger (TB) classifiers [43].

Radiomics has been widely applied in clinical diagnosis, disease classification, prognosis prediction, treatment decision-making, and other fields [11]. Its essence is to directly map data to clinical outcomes. However, in most studies, the biological mechanisms underlying the indirect relationships that can be predicted by radiomics remain largely unexplained. The exploration of biomarkers in radiomics is almost entirely data-driven, and the visual properties of the considered features ensure that the research results are still relatively biased toward understanding the physical mechanisms [44]. Our current research focuses on the direct relationship between the radiomics data and the clinical results, to explore which radiomics features are related to the prognosis of pancreatic cancer patients with hepatic metastasis after chemoimmunotherapy. So far, there are four main biological correlations and methods used for the biological basis of radiomics, including expression data, protein expression from immunohistochemistry staining, microscopic histologic textures, and physiologic tumor habitats [44]. In subsequent research, we will focus on revealing the biological significance of radiomics by combining the results of other biological studies such as histopathology, genetics, and proteomics.

## Conclusion

- ORR exhibits no significant correlation with either PFS or OS.
- The nomogram, which combines radiomics features with clinical data, respectively, outperforms the radiomics and clinical models in terms of prognostic prediction.
- The predictive model derived solely from clinical data demonstrates efficacy in forecasting PFS and ORR, but lacks the ability to predict OS.
- Radiomics features hold predictive value for PFS, OS, and ORR. The predictive performance of multi-lesion feature radiomics models surpasses that of models relying on single-lesion features. Furthermore, the utilization of multi-sequence features yields superior predictive performance compared to single-sequence feature radiomics models. The radiomics features extracted from pancreatic and hepatic lesions and calculated by volume weighting, jointly constructed by T2 and DWI sequences, have good predictive performance for prognosis.

### Supplementary Information

Below is the link to the electronic supplementary material.

Supplementary file1 (DOCX 0 KB)
