# Correction to: The radiomics nomogram predicts the prognosis of pancreatic cancer patients with hepatic metastasis after chemoimmunotherapy

**DOI:** 10.1007/s00262-024-03739-w

**Published:** 2024-07-05

**Authors:** Wenxin Lu, Guangyu Wu, Xianyuan Miao, Jingyu Ma, Yanling Wang, Haiyan Xu, Daiyuan Shentu, Shengbai Xue, Qing Xia, Yu Wang, Liwei Wang

**Affiliations:** 1 Department of Oncology, Ren Ji Hospital, Shanghai Jiao Tong University School of Medicine, Shanghai, 200127 China; 2 Department of Radiology, Ren Ji Hospital, Shanghai Jiao Tong University School of Medicine, Shanghai, 200127 China; 3 Department of Oncology, Ning Bo Hangzhou Bay Hospital, Ningbo, 315336 China; 4 State Key Laboratory of Systems Medicine for Cancer of Shanghai Cancer Institute, Ren Ji Hospital, Shanghai Jiao Tong University School of Medicine, Shanghai, 200127 China

**Correction to: Cancer Immunology, Immunotherapy (2024) 73:87** 10.1007/s00262-024-03644-2

In the original version of this article, in the ‘’Materials and methods’’ section, second sentence under the heading ‘’Patients**’’,** there are some mistakes in the description of these three sets. Second sentence which previously read

74 patients diagnosed with PDAC and hepatic metastasis who visited Renji Hospital (Shanghai, P.R. China) from June 2019 to November 2021 were selected and randomly assigned to the training set (*n* = 52) and the validation set (*n* = 22) according to a 7:3 ratio. 31 patients enrolled in the "Prospective one arm exploratory clinical trail on the efficacy and safety of PD-1 antibody SHR-1210 combined with AG in the first-line treatment of metastatic pancreatic cancer" (RenJi Hospital, ClinicalTrials.gov Identifier: NCT04181645) from November 2019 to March 2021 were selected and included in the testing set.

Should have read

52 patients enrolled in 2 clinical trials in Renji Hospital (ClinicalTrials.gov Identifer: NCT04181645, NCT04674956) from August 2019 to March 2022 were selected and included in the training set. 53 patients diagnosed with PDAC and hepatic metastasis who visited Renji Hospital (Shanghai, P.R. China) from June 2019 to November 2021 were selected and randomly assigned to the validation set (n = 22) and the testing set (n = 31).

In Fig. 1 showed wrong description of 3 sets of patients. The Fig. 1 should have appeared as shown below.

**Fig. 1:**
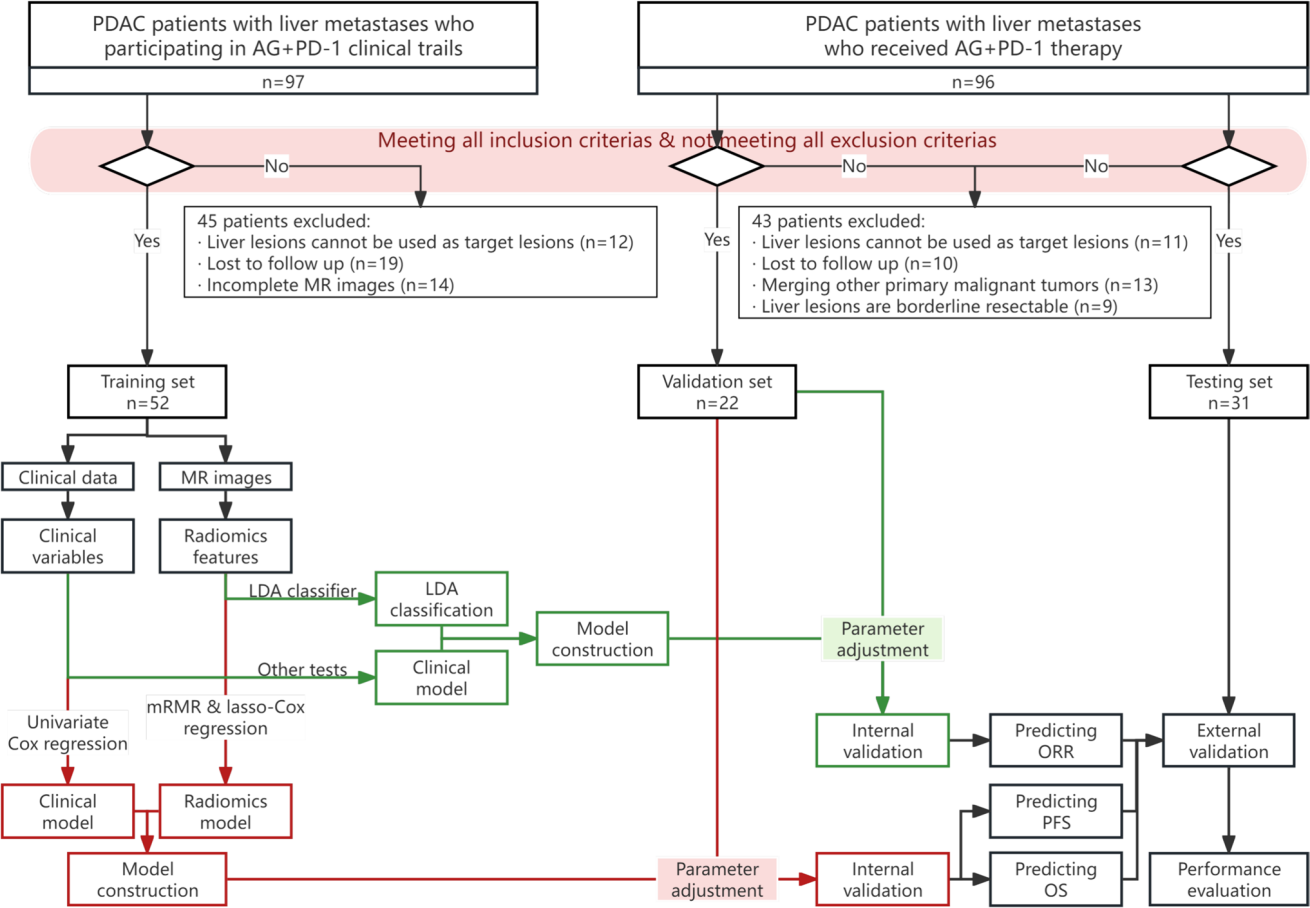
The flowchart of this study

In the second sentence under the section ‘’Evaluation of radiomics features’’, "95 Glszm features" was incorrect. There are 95 Glszm and Glrlm features in total. So it should be “33 Glszm features”.

Second sentence which previously read

411 features in DWI sequences were selected, containing 9 original shape-based features, 160 first-order features, 147 Glcm features, 62 Glrlm features and 95 Glszm features.

Should have read

411 features in DWI sequences were selected, containing 9 original shape-based features, 160 first-order features, 147 Glcm features, 62 Glrlm features and 33 Glszm features.

