# Supplementary material for: The radiomics nomogram predicts the prognosis of pancreatic cancer patients with hepatic metastasis after chemoimmunotherapy
Source: Cancer Immunol Immunother. 2024 Mar 30;73(5):87. doi: 10.1007/s00262-024-03644-2 (PMC10981596; doi:10.1007/s00262-024-03644-2)
Supplement: Supplementary file 1 — Supplementary file1 (DOCX 0 KB) [file 262_2024_3644_MOESM1_ESM.docx]

**Supplementary Materials for “The radiomics nomogram predicts the prognosis of pancreatic cancer patients with hepatic metastasis after chemotherapy combined with immunotherapy”**

**Supplementary Material 1**


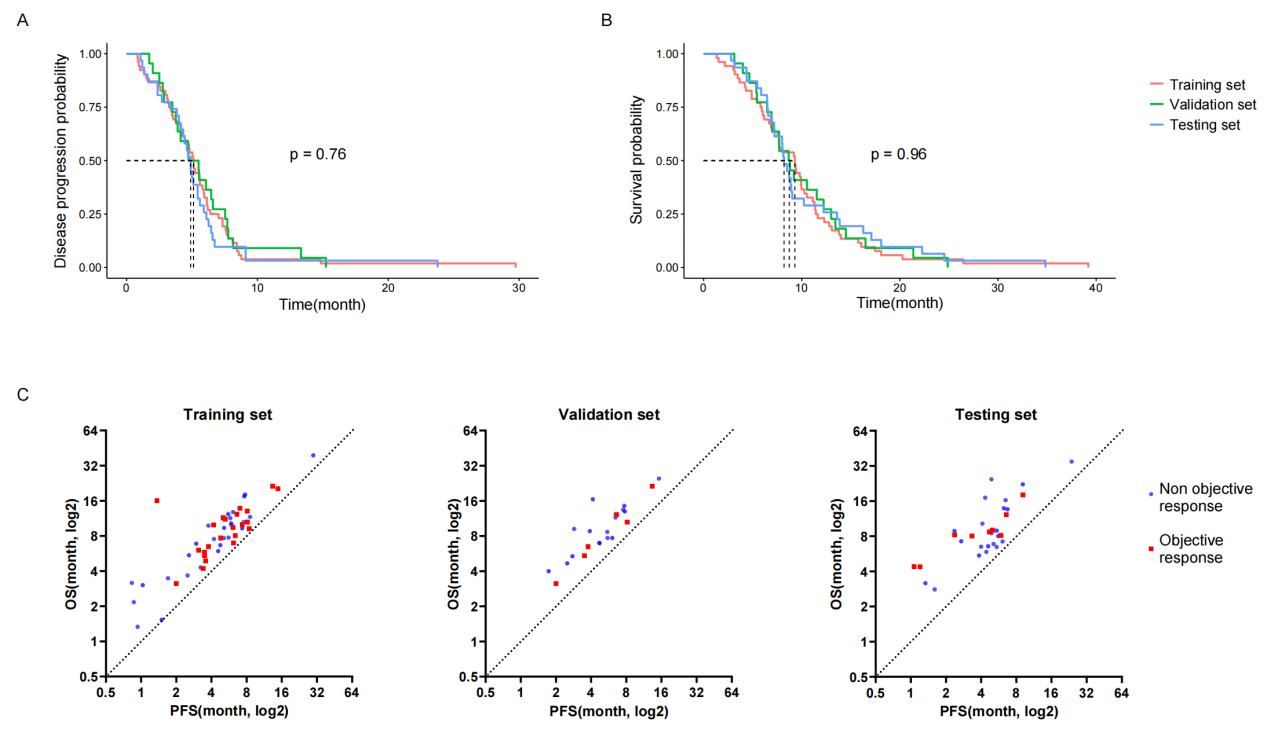


**Figure S1. Distribution of PFS, OS and ORR in training set, validation set and testing set.** The differences of PFS **(A)** and OS **(B)** in training set, validation set and testing set are not statistically significant. **(C)** The ORR is not correlated with PFS or OS whether in training set, validation set or testing set.


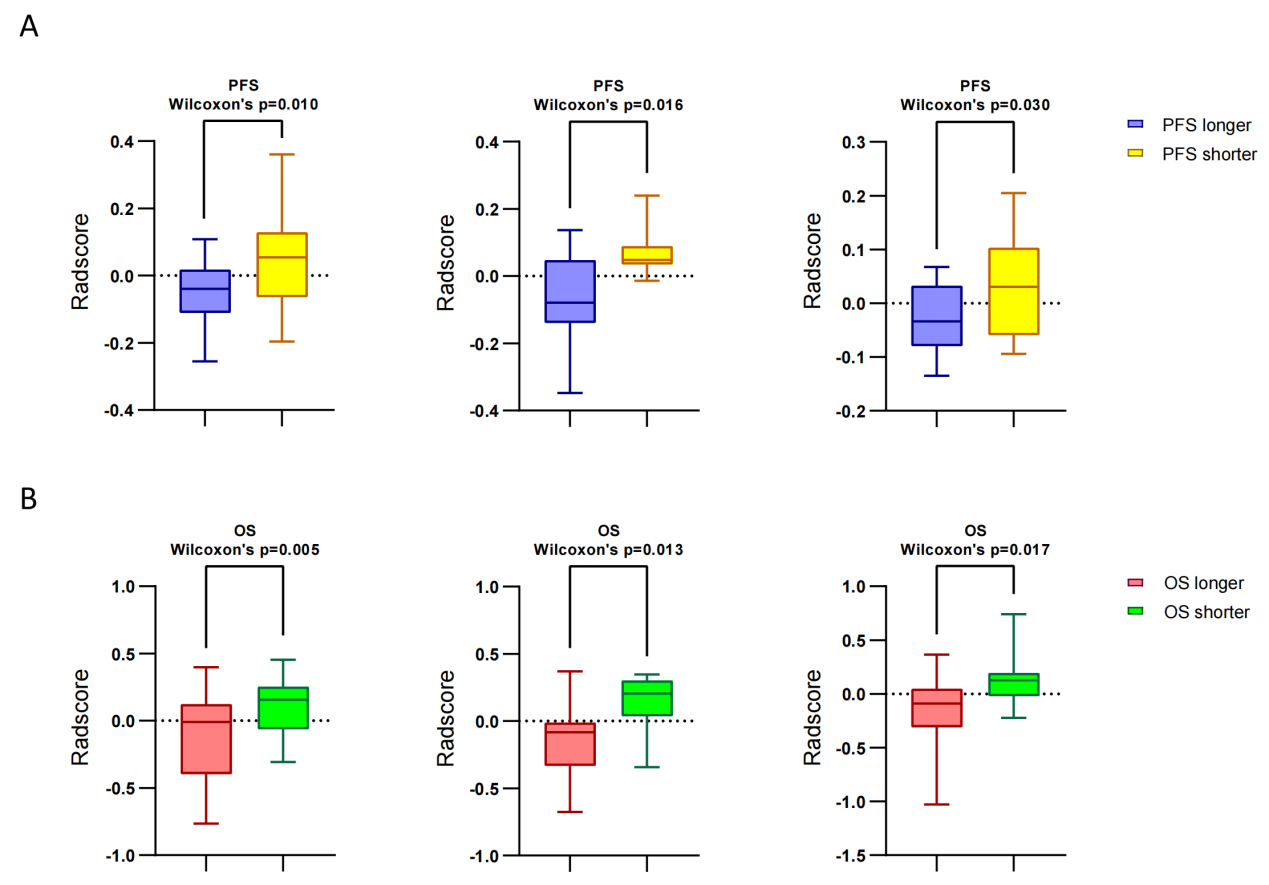


**Figure S2. Radscore of PFS and OS radiomics model in training set, validation set and testing set.** Radscore performs well in predicting PFS and OS in all sets. Groups indicates patients with PFS/OS exceeding the median and those without exceeding the median. **(A)** Distribution of radscores for PFS in training set, validation set and testing set. **(B)** Distribution of radscores for OS in training set, validation set and testing set.

**Supplementary Material 2**

The formula of radscore for PFS:

*Radscore = DWI-log-sigma-3-0-mm-3D_glrlm_GrayLevelNonUniformity *0.387844460*

*+T2-original_shape_Roundness *0.148049775*

*+T2-squareroot_glcm_Imc1 *0.177956325*

*-DWI-original_glcm_InverseVariance *0.370454455*

*-DWI-logarithm_glszm_ZoneVariance *0.078461145*

*+T2-log-sigma-3-0-mm-3D_glszm_IntensityVariability *0.078461145*

*+T2-log-sigma-3-0-mm-3D_glszm_LowIntensitySmallAreaEmphasis *0.019462710*


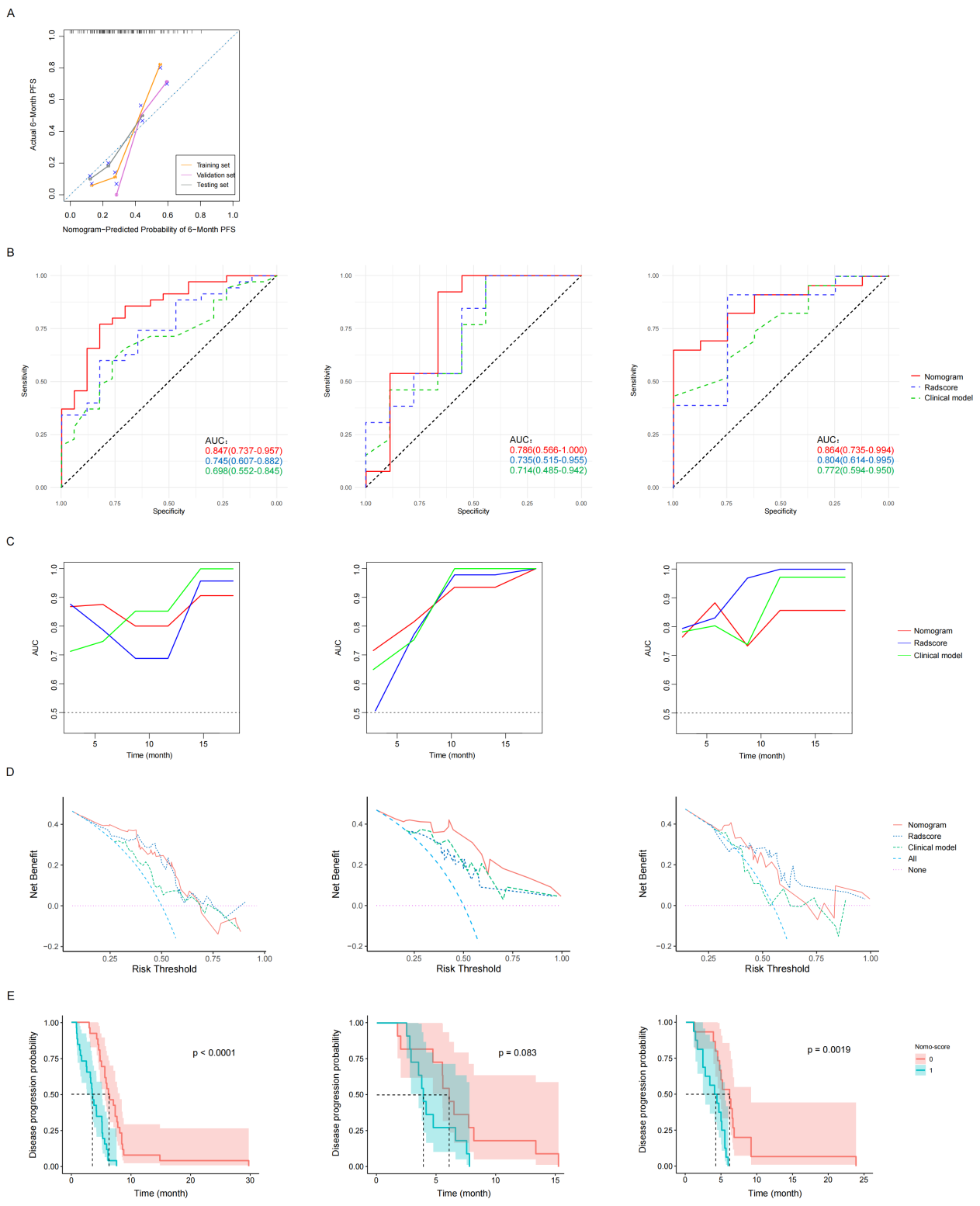


**Figure S3. Evaluation of nomogram for predicting PFS.** Nomogram performs well in predicting PFS, outperforming radiomics and clinical model. **(A)** The calibration curves presenting good consistency between the nomogram-predicted probability of 6−Month PFS and the actual 6−Month PFS. **(B)** The ROCs representing the prediction performance of the nomogram, radscore, and clinical model for 6-month PFS in training set, validation set and testing set. **(C)** The time-AUC curves representing the time-correlated prediction performance of the nomogram. **(D)** The DCA curves showing the predictions of the nomogram, radscore, and clinical model for 6-month PFS in training set, validation set and testing set. **(E)** Kaplan-Meier analysis between patients with lower nomogram score (group 0) and higher nomogram score (group 1) in training set, validation set and testing set, p value is calculated from Log-Rank test.


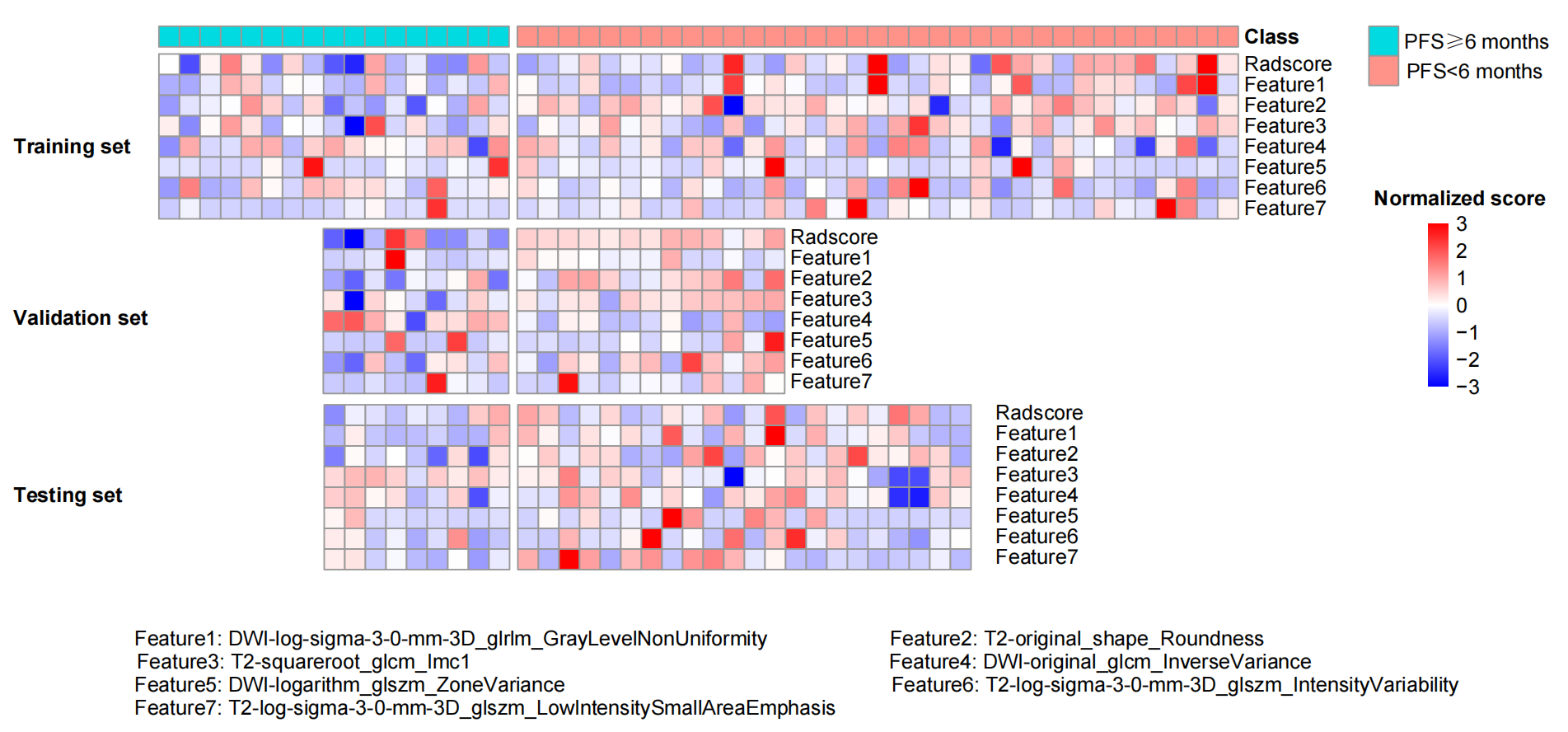


**Figure S4:** Heatmap for PFS: Constructed from radscore and the 7 chosen features of all patients.

**Supplementary Material 3**

The formula of radscore for OS:

*Radscore = DWI-log-sigma-3-0-mm-3D_glrlm_GrayLevelNonUniformity *3.423290e-02*

*+T2-original_shape_Roundness *2.704273e-01*

*+T2-log-sigma-3-0-mm-3D_glszm_ZoneEntropy *3.834040e-02*

*-DWI-original_glcm_InverseVariance *2.593034e-05*

*-T2-original_shape_Compactness2 *1.441038e-02*

*-DWI-logarithm_glszm_ZoneVariance *1.883117e-01*

*+T2-log-sigma-3-0-mm-3D_glszm_LowIntensitySmallAreaEmphasis *5.095323e-03*


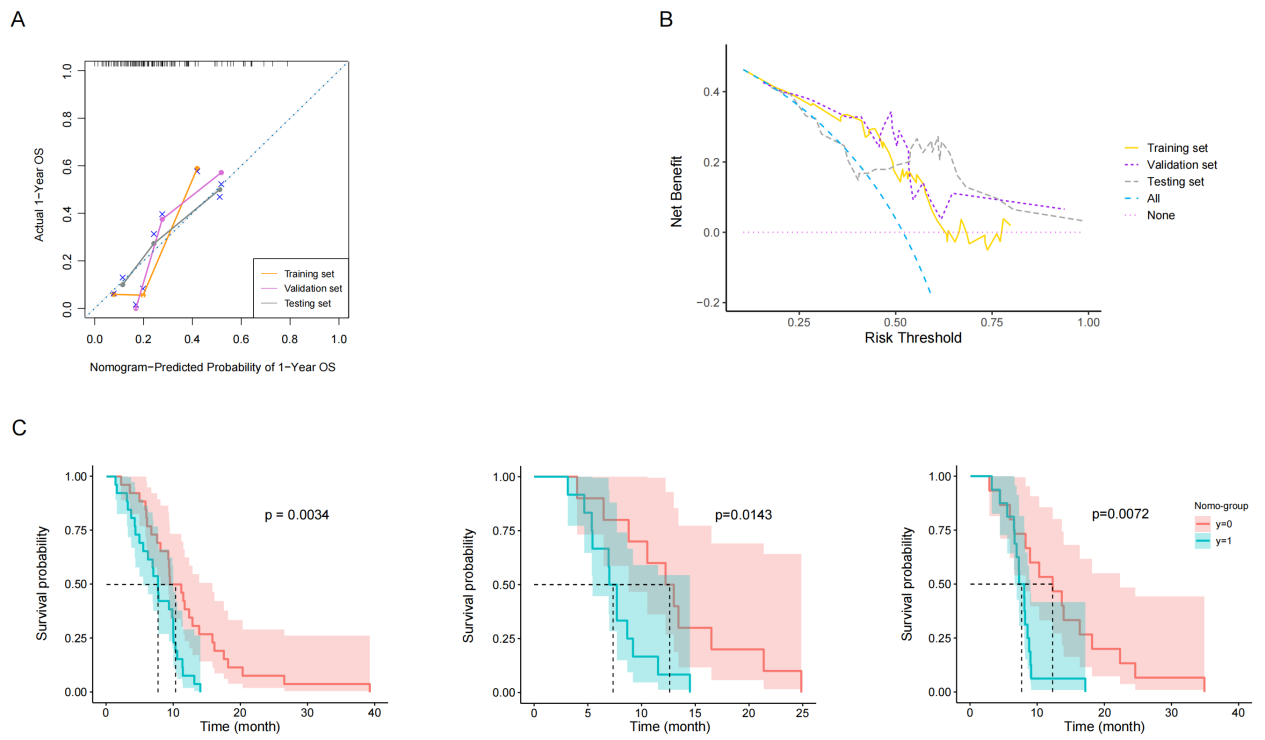


**Figure S5. Evaluation of nomogram for predicting PFS.** Nomogram performs well in predicting OS. **(A)** The calibration curves presenting good consistency between the nomogram-predicted probability of 1-Year OS and the actual 1-Year OS. **(B)** The DCA curves showing the prediction performance of the nomogram for 1-year OS. **(C)** Kaplan-Meier analysis between patients with lower nomogram score (group 0) and higher nomogram score (group 1) in training set, validation set and testing set, p value is calculated from Log-Rank test.


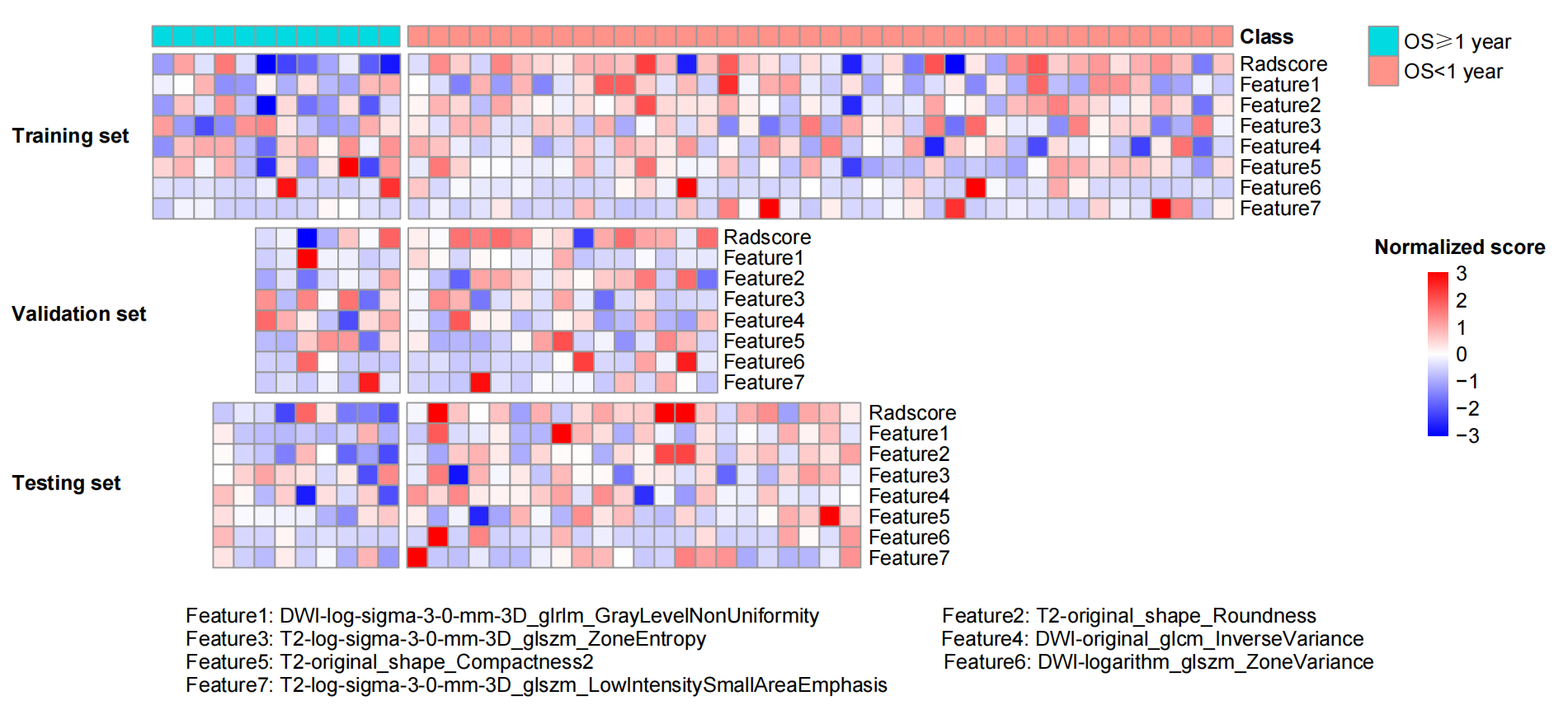


**Figure S6**: Heatmap for OS: Constructed from radscore and the 7 chosen features of all patients.

**Supplementary Material 4**


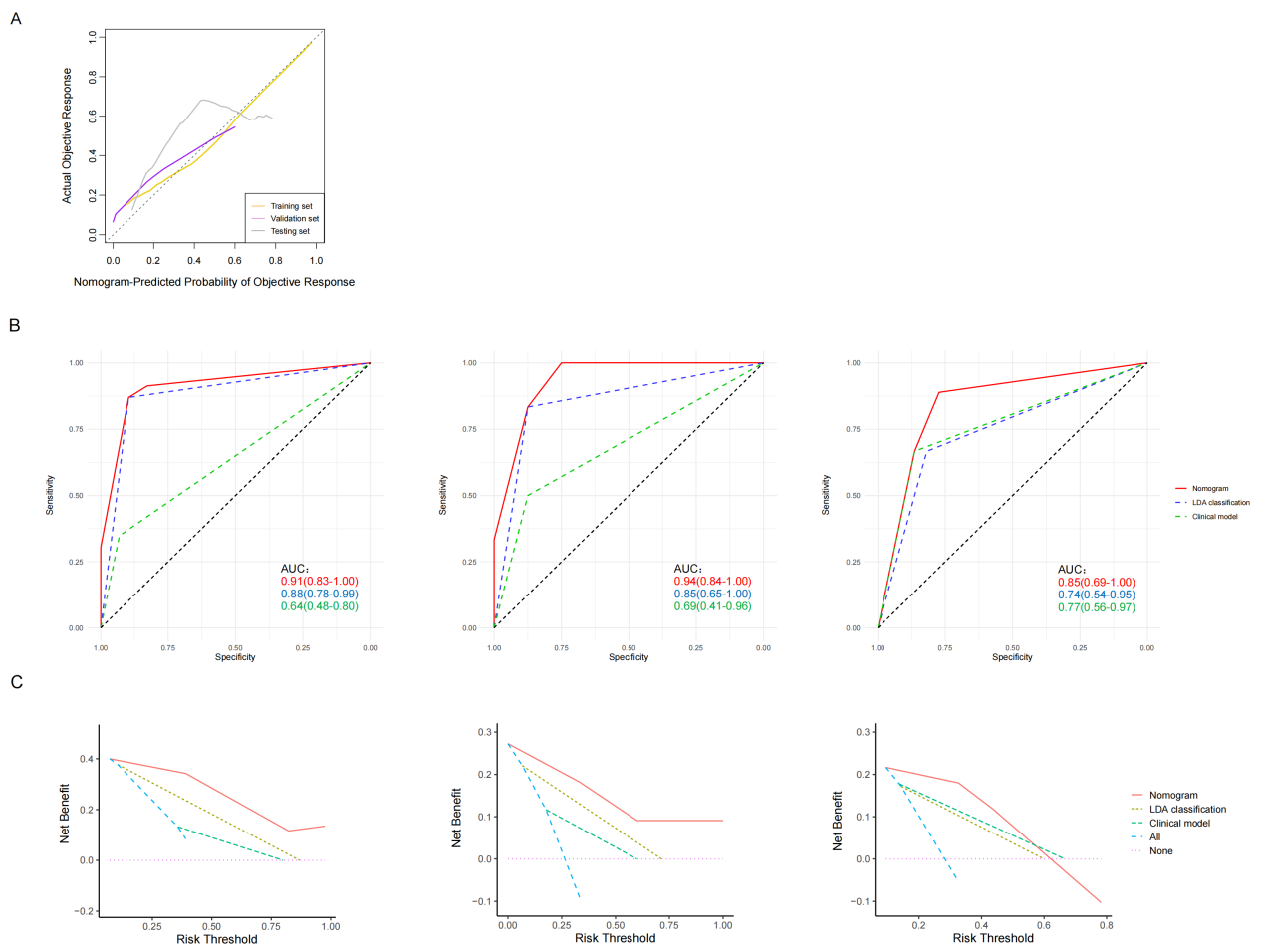


**Figure S7. Evaluation of nomogram for predicting ORR.** Nomogram performs well in predicting objective response, outperforming LDA classification and clinical model. **(A)** The calibration curves presenting consistency between the nomogram-predicted probability of objective response and the actual objective response. **(B)** The ROCs representing the prediction performance of the nomogram, LDA classification, and clinical model for objective response in training set, validation set and testing set. **(C)** The DCA curves showing the predictions of the nomogram, LDA classification, and clinical model for objective response in training set, validation set and testing set.

**Table S8. The detailed MRI protocol.**

| Sequence | DWI | T2-Weighted  Imaging | T2-Weighted  Imaging | T1-Weighted Imaging | Contrast-enhanced Dixon |
| --- | --- | --- | --- | --- | --- |
| Scan plane | Axial | Axial | Coronal | Axial | Axial |
| TR (ms) | 2100 | 3650 | 363 | 3.97 | 3.97 |
| TE (ms) | 51/80/110/140/180/200 | 92 | 96 | 1.29 | 2.52 |
| FOV (mm^2^) | 380*283 | 380*380 | 400*400 | 380*308 | 380*308 |
| Slice Thickness/Gap(mm) | 4/1 | 4/1 | 3/0 | 3/0 | 3/0 |
| Acquisition matrix | 268*200 | 384*384 | 320*256 | 320*182 | 320*182 |
| Bandwidth (Hz/pixel) | 710 | 723 | 710 | 1040 | 1040 |
| Respiratory Control | Trigger | Trigger | Breath-hold | Breath-hold | Breath-hold |
| other | b-values=0/800(s/mm^2^) | fat saturation | / | / | / |

**Supplementary Material 5**

**
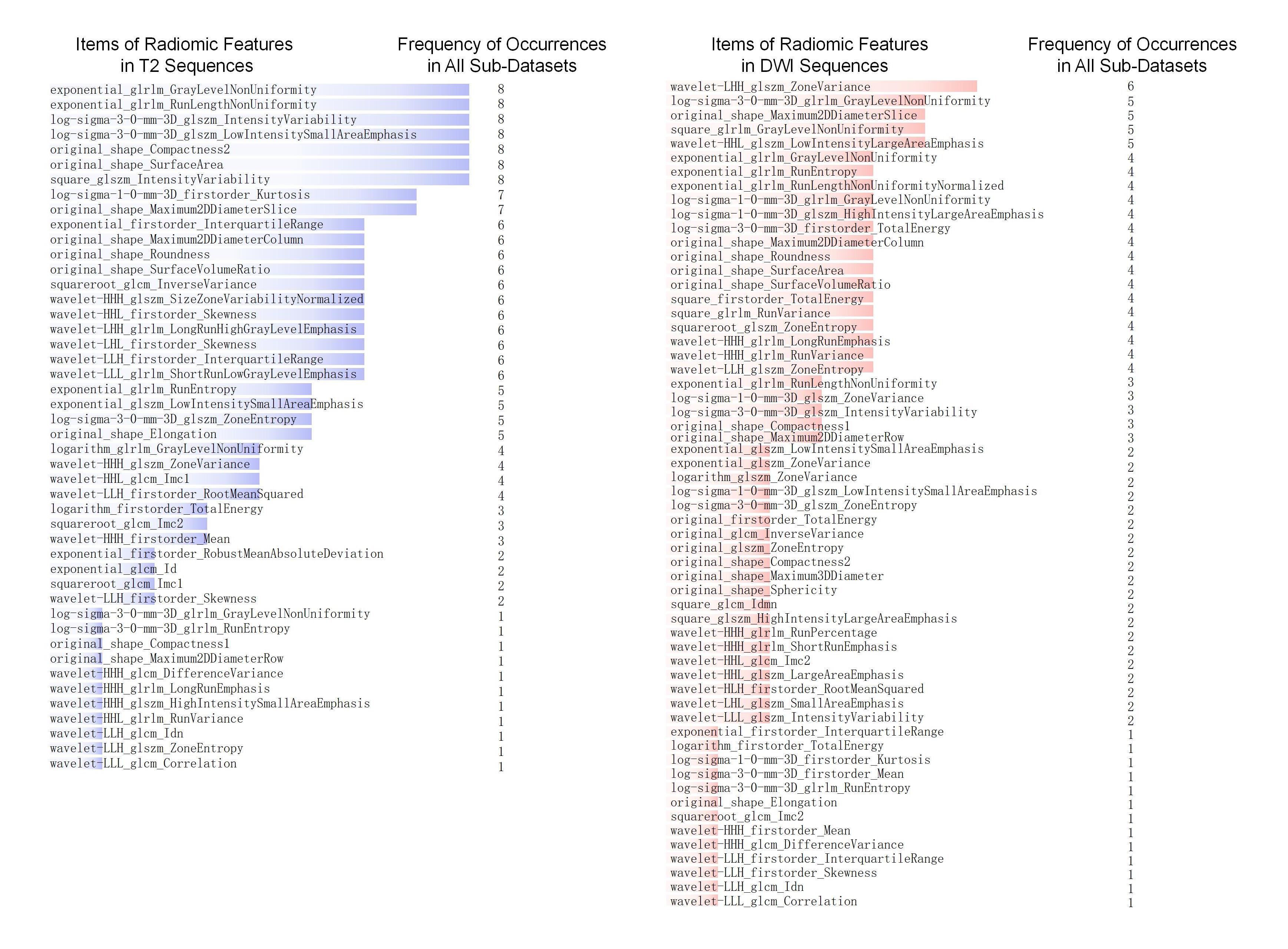
**

**Figure S9. Frequency list of all features retained by mRMR algorithm in 6 datasets.** 6 datasets were screened using the MRMR method for predicting PFS and OS, with 30 features retained each time, resulting in a total of 12 30-feature-subsets and 360 features being retained. Each 30-feature-subset is inconsistent, with some features being conserved and appearing repeatedly among 12 subsets. Among the T2 and DWI sequence features, the feature with the highest frequency appeared 8 and 6 times in these 12 subsets. After removing duplicate features, there were 46 and 59 non repetitive features from T2 and DWI sequences respectively.


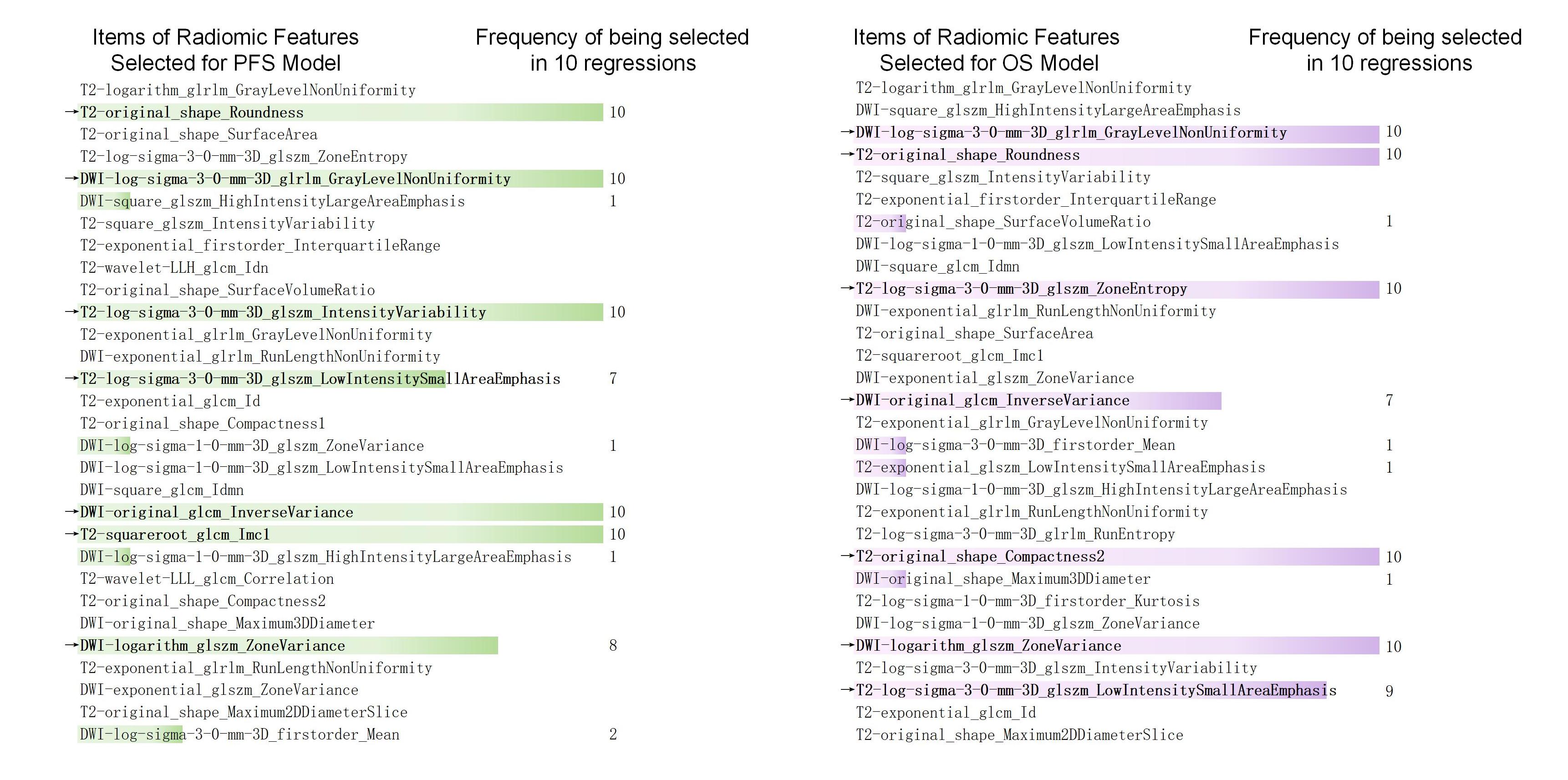


**Figure S10. Frequency list of all features selected by Lasso regression in PFS and OS radiomics models.** Respectively in PFS and OS models, 10λ values were put out by running the cv.glmnet function 10 times, and regression modeling were conducted 10 times respectively according to the λ values. Some features were conserved and selected repeatedly among 10 regressions. 7 features were respectively chosen to construct the T2 and DWI models, which all showed high frequency of being selected.

→: Features that we ultimately chose to construct radiomics models for PFS and OS.

**Supplementary Material 6**


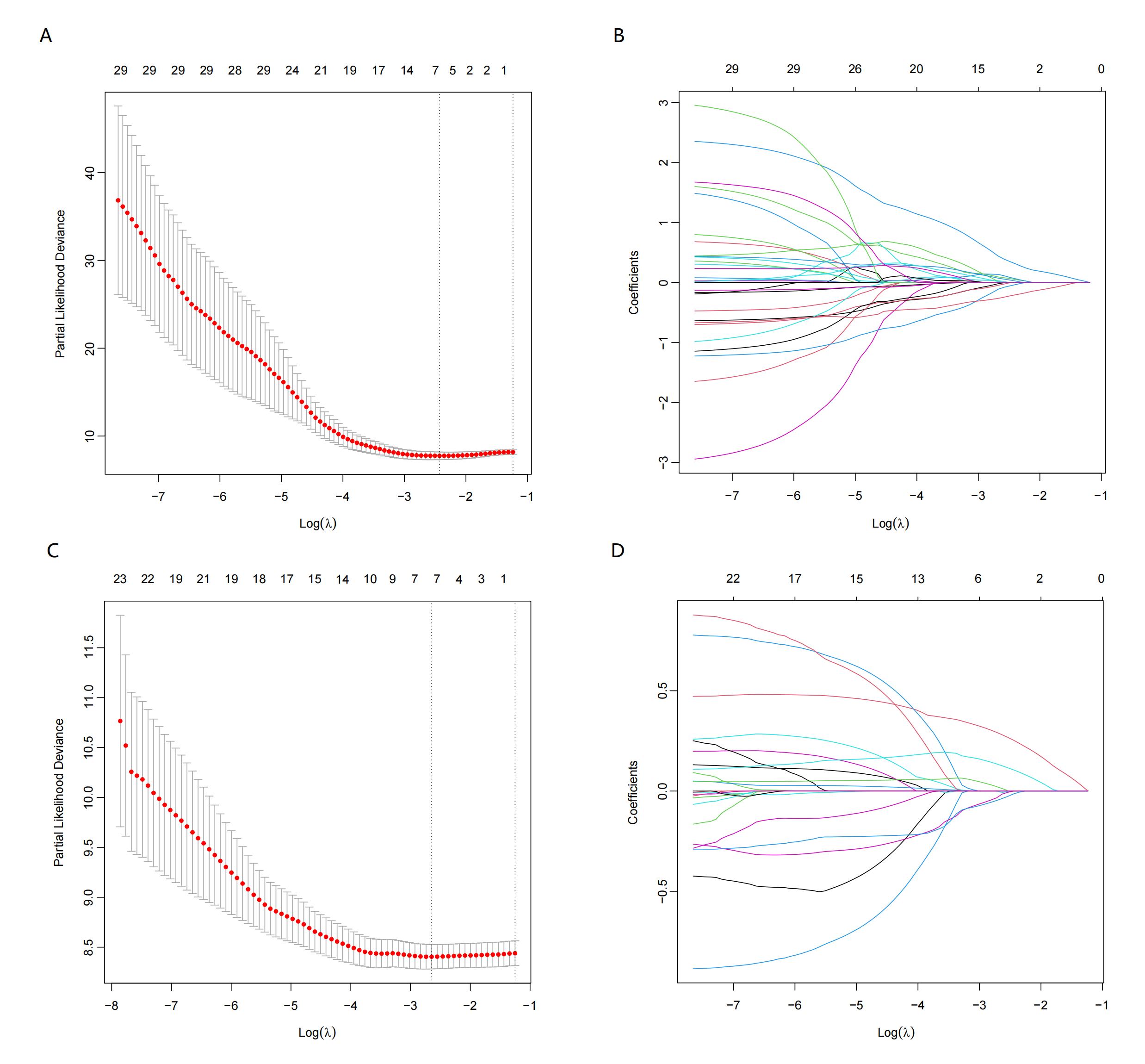


**Figure S11. Lasso-Cox regression analysis.** Partial likelihood deviance in **(A)** PFS model and **(C)** OS model was plotted versus log (λ). The left vertical dotted line indicates the λ value with the minimum error (i.e. lambda.min), and the right vertical dotted line indicates the λ value which obtains the simplest model within a variance range of lambda.min (i.e. lambda.1se). Lasso coefficient profiles of selected features in **(B)** PFS model and **(D)** OS model associated with log(λ).
